# Steps towards a New Humanism in the Mental Health Disciplines - An Interview with Tim Ingold

**DOI:** 10.1007/s12124-024-09877-1

**Published:** 2025-01-03

**Authors:** Bård Bertelsen, Odd Kenneth Hillesund, Tore Dag Bøe, Per Arne Lidbom, Rolf Sundet, Tim Ingold

**Affiliations:** 1 University of Agder, Grimstad, Norway; 2 University of South-Eastern Norway, Drammen, Norway; 3 University of Aberdeen, Aberdeen, United Kingdom

**Keywords:** Mental Health, Anthropology, Undergoing, Correspondence, Care, Tim Ingold

## Abstract

This interview article explores how British anthropologist Tim Ingold’s work can inspire innovation in mental health and the psy disciplines. Ingold critiques dominant biomedical and individualistic approaches, arguing for the importance of caring attentiveness and abolishing dichotomies like those between surface and depth, when engaging with people to understand and assist them. Instead, he suggests viewing human existence as correspondences with environmental, social, and relational others. The interview highlights the concept of “doing-undergoing,” proposing that care is a reciprocal, relational process. Ingold’s ideas suggest a shift towards practices that engage directly with the world and promote attentiveness to human and more-than-human relations. The article encourages practitioners, educators and students of mental health disciplines to rethink traditional models and adopt more humane approaches.

## Introduction

In this article, we explore how the ideas of the British anthropologist Tim Ingold can be used to inspire innovation in the mental health field. The central part of the article consists of an interview with Ingold. The interview was conducted by the five authors, who are academics with either current or previous experience in mental health practice, family therapy, and psychotherapy.

Ingold is an Emeritus Professor of Social Anthropology at the University of Aberdeen and is considered one of today’s most widely read and influential anthropologists. He presents a holistic and integrative approach to understanding human life and its relation to the environment. Ingold’s ideas on ecological approaches, human-animal relations, and the anthropology of lines have fostered new ways of thinking in diverse fields.

### Turning towards Reality in the Psy Disciplines

In a recent article, Brinkmann et al. (2023) draw on Ingold’s work to critically examine the prevailing biologizing trends in conceptions of mental health, arguing for an alternative, non-reductive biologization grounded in ecological and contextual understandings. They emphasise the importance of considering human beings within their environmental niches and social contexts rather than viewing mental health issues primarily through neurobiology and genetics focused narrowly on the individual. Hence, they propose a shift towards an ecological perspective for the psy sciences (psychology, psychiatry, and psychotherapy), aimed at understanding mental disorders as situational and relational responses rather than intrinsic dysfunctions. This approach encourages interventions that consider both the individual and their environment, promoting a more holistic understanding of mental health that integrates biological, psychological, and social dimensions.

Such a form of realism, which is not linked to scientific realism but is more accurately covered by a term such as ethical or existential realism (Bøe, 2021), is also at the heart of the ongoing research initiative in mental health and psychotherapy that we have named Return to Reality (Bøe et al., 2024). The initiative organises a variety of explorations into how to shift from theoretical models that distance and obscure the ethical demands and experiences of individuals towards more reality-sensitive approaches that emphasise direct encounters with the world and with others. Ultimately, the project calls for more humane and contextually aware approaches to mental health.

### Interview as Scientific Inquiry

Although uncommon as a form of scientific inquiry, the interview format offers a uniquely reality-oriented approach to exploring how a particular thinker’s ideas can illuminate and expand the scope of a field such as mental health. Unlike more essayistic forms or literature reviews, the interview provides a dynamic and conversational platform where one thinker is directly invited to engage with the concepts and ideas under study. The format fosters an immediate and relatable connection between the interviewers, the thinker whose ideas are being explored, and the reader, keeping the text grounded in real-world contexts and highlighting the practical implications of the ideas under discussion. In addition, the dialogic interactional form exemplifies the relational and situational understanding advocated by Ingold. This dialogical form is also essential to the Return to Reality initiative of which this article is part.

Rather than offering a roadmap to using Ingold’s ideas for improving practice, this interview aims to inspire discussions on how the mental health field can benefit from engaging with anthropology. However, we do not mean to advocate an “anthropologisation” of mental health care. Instead, we hope the conversation may inspire further rethinking of practice among mental health practitioners, educators, and researchers.

## The Interview

The interview was conducted in written form during the spring of 2024. Our questions and Ingold’s responses are presented as formulated in the interview setting. To help the reading, we have added sub-headings indicating the central ideas or concepts being discussed.

### What is a Life?

*A possible reading of your work is that it is a continuing argument for a different conception of a life than the essentialist or naturalistic figure of the person that a medicalised framework of mental health «treatment» presupposes. Can you elaborate a bit on what you think a life is?*

Let me begin by explaining what I think a life is *not*. It is not the chronological list of steps taken, milestones passed, and achievements recorded, that adds up to what we would now call a curriculum vitae. A lived life continually weaves in and around the events that the curriculum vitae tots up, neither beginning nor ending anywhere in particular, but always overtaking itself. In life, we do not write out a programme or script that has been laid down in advance, but rewrite the script as we go along. Life is improvisation. And as with all improvisation, it is not done in a vacuum, but rather in response to all the other lives that are going on around us, human and other-than-human.

*You draw on Dewey*,* who distinguishes between doing and undergoing. Can you elaborate on what you make of that distinction? What would some consequences of thinking of living as doing undergoing be for professional caring practices?*

Conventionally, doing and undergoing are opposed as active to passive. In the active voice of the verb, you do something. In the passive voice, something is done to you. But this construction leads us to think of the undergoing as contained within the doing. My understanding of Dewey is that he turns this relation upside down, through a focus on experience. In experience, he argues, undergoing always overflows doing. In everything you do, you are personally transformed in the doing of it, which means that you enter every next thing as a slightly different person. That’s how and why we continually create ourselves as we go along. In this sense of self-creation, in relation with others, the undergoing is not passively suffered but actively done. That’s what I mean by doing undergoing.

Because we do undergoing in relation with others, to whom we simultaneously attend and respond, it is also a practice of care. This does have implications for the way we think about care. Once again, our conventional understanding of care is locked into the opposition between active and passive voices. If you are the agent, you do care. If you are the patient, care is done to you. But with doing undergoing, it is impossible to care without at the same time being cared for: your care for another implies the other’s care for you. It is like a grown-up holding a child by the hand. We would typically describe the adult as caring for the child. Yet we could just as well say that the child is holding the adult by the hand. So, who is caring then? Each, in the doing undergoing of holding hands, is at once caring and cared for.

*You say that we*,* as humans*,* don’t just live our lives - we lead them (*Ingold, 2018b, p.*20). And you argue that the difference between living a life and leading a life is attention. Can you elaborate on this and how it relates to caring or healing practices?*

A life that is led is one that always stretches out towards others: this stretching out is the literal meaning of attention (from the Latin *ad*, ‘towards’, plus *tendere*, ‘to stretch’). I’ve also described this as a process of longing. We long for people, and for things, and it is this that draws us along, along the path of doing undergoing. It is equivalent to what Hannah Arendt called *amor mundi*, ‘love for the world’. Leading life enacts this love.

This implies, I suppose, that *amor mundi* is also a condition of care. It is certainly a condition of education, which was Arendt’s point. Her argument was that the responsibility of the educator is to introduce new people into old ways – that only by doing so is there hope of renewal for generations to come (Arendt, 1961). Maybe the same argument could be extended to care. It would mean thinking of care, and of healing, as a process of renewal or rejuvenation. Instead of drawing a line under the past, rejecting it in anticipation of better times ahead, it would mean recognising the good in the life already undergone, as affording a way to carry on.

### Thinking and Acting with

*In your book Making* (Ingold, 2013a), *you characterise anthropology as “transformational” and fundamentally different from ethnography*,* which you describe as “documentary”. You emphasise the importance of doing “anthropology with” rather than “anthropology of”. In line with this*,* your approach to anthropology focuses on “knowing from the inside*,*” emphasising the need to “grow into” things and let them “grow in you” to truly understand them. Anthropologists*,* you say*,* should “study* with *people […] and hope to learn* from *them”* (Ingold, 2013a, p. 2, emphasis in original).

*In Making (*Ingold, 2013a*)*,* you position ethnography as a documentary and hermeneutic enterprise concerned with the detailed study and description of human practices within specific cultural contexts. You characterise anthropology*,* by contrast*,* as a practice of speculative imagination ultimately concerned with finding ways of sustaining liveable lives together with the more-than-human world around us.*

*Do you think your distinction between ethnography and anthropology reaches wider than your own academic field? Do you think it points to a more general or fundamental debate?*

My aim is indeed that it should reach beyond anthropology, though whether it has done so to any significant extent is hard to say. To fellow anthropologists who accuse me of tilting at windmills – we all have an intuitive, in-house understanding of what we do and have no need to formalise it – I point out that it is precisely when we move *beyond* the borders of the discipline and are challenged to explain what we do to others, that it is so important to distinguish anthropology from ethnography. Only then can we counter the common preconception that anthropologists are only there to tell stories about other people, about what they do and think. It is a preconception that leaves us without a voice of our own, resulting in our exclusion from the big public debates dominated by economists, psychologists, historians, philosophers, and others, in which a strong anthropological voice is badly needed.

Behind the distinction, there is indeed a more fundamental debate. This is about how we move on from the humanism of the Enlightenment, with its commitment to human exceptionalism, progress and emancipation from nature. Everyone agrees that this has run out of road. For many, the alternative lies in a doctrine of posthumanism. But due to the complicity of humanism in the world-historical project of colonisation, the issue of posthumanism tends to get mixed up with the wider debate about decolonisation. I believe that despite the protests of many of my colleagues to the contrary, the project of ethnography – writing about people based on data extracted from them – is still intrinsically colonial. I see my advocacy of anthropology, as a way of studying *with*, as a decolonial move. But I also see it as offering an alternative to posthumanism; rather a *new humanism*, which remains human-centred, yet takes as its starting point the positioning of humans within the fold of a world of relations and processes, for the flourishing of which they bear an exceptional responsibility. This is something I am still working on, as we speak, so I cannot yet be clearer than that.

*What could the consequences of providing more room for the kind of thinking with (as opposed to accounting for) people be for societal institutions like mental health services? Do you think a turn from retrospective explanatory knowledge structures to a more imaginative or speculative and prospectively transformative approach is possible – and wise – to work for in the mental health care sector?*

I am not really competent to answer this question. I am however conscious of how the discourse of ‘mental health’ seems to be largely concerned with putting ways of thinking and of being that diverge from the norm into explanatory or interpretative ‘contexts’, such that they can be understood and accounted for. But this is also a way of making them invisible. Embedded in their contexts, divergent minds are also removed from our presence, so that they cease to pose a direct challenge. We need no longer be troubled by them.

Much of contemporary art, I think, is trying to do the reverse – to dis-embed the divergent, to bring it back into presence, to trouble our perceptions of normality. Maybe that’s where we should look for an alternative, more transformative approach. But this would also mean rescuing artistic interventions from the clutches of art critics and historians who, just like mental health professionals, seem intent on shoving artworks back into their contexts once again!

### Intention vs. Attention

*From The Perception of the Environment (2000) onwards*,* you often return to attention as a critical element for understanding the interconnectedness of perception*,* materiality*,* and life. Attention is not merely a cognitive process but a holistic engagement that connects humans to their environments*,* other beings*,* and the materials they interact with. This relational approach to understanding human life emphasises the importance of attentiveness to the ongoing formation of the self and the world*,* suggesting that how we attend to our surroundings shapes our knowledge*,* experiences*,* and ethical relations. Through practices of attention*,* individuals can cultivate a responsive and responsible way of living within the world. Sometimes*,* you link attention to wisdom and contrast this with knowledge. For example*,* in your book Anthropology - Why it Matters (*Ingold, 2018a*)*,* you write: “The more we take refuge in the citadels of knowledge*,* the less attention we pay to what is happening around us. Why bother to attend*,* we say*,* when we already know? To be wise*,* on the contrary*,* is to venture out into the world and take the risk of exposure to what is going on there. It is to let others into our presence*,* pay attention*,* and care” (p. 9).*

*In our field of mental health care*,* knowledge is everywhere*,* and every action and measure should be knowledge- or evidence-based. In the education of future mental health practitioners*,* intentions are often seen as things that practitioners bring into the conversation from somewhere else. Attention requires us to set these aside and open up to what is before us.*

*As an anthropologist*,* how do you relate to this distinction between intention and attention? How do you* do *attention?*

Anthropologists do attention by way of what they call ‘participant observation’. This is really another expression for attentive practice. It is often said that participant observation is a contradiction in terms: after all, how can you participate and observe at the same time? In one you are seemingly in the midst of things; in the other you are looking on from a position of detachment. Critics say that participant observation is like asking you to swim in the river and stand on the banks at the same time. But this criticism, I think, rests on a confusion of observation with objectivity. The protocols of scientific objectivity do indeed demand that the investigator is immunised (by ‘methodology’) from any influence arising from sensory contact with the objects of their investigation. But observation, quite to the contrary, is a way of following what is going on, coupling the movement of one’s attention to a movement in the world. This coupling is what we mean by participation. It follows that there can be no observation without participation. And it is precisely when the things with which we study begin to tell us how to observe (as people do, in the course of anthropological fieldwork) that observation goes beyond objectivity.

*What are some of the significant challenges to attentive practice* *?*

Two, mainly. One is that you have to learn to notice things, to pay attention to them. It is a skill, and it takes time to acquire. The other is that it is impossible to attend to everything at once. If your attention is caught by one thing, it is liable to miss others. And for anthropologists, who want to catch everything that is going on, this can be a challenge.

*How do we hold on to attention and wisdom within institutional systems that are increasingly fine-tuned to eliminate precisely the kinds of uncertainties that are prerequisite for existence?*

You tell me! If only we had the answer. I can only say, from my personal experience of university teaching, that it is a never-ending struggle. It would be too simple to argue that we should strive to bring down the institutions. Today, we are seeing a widespread loss of faith in once respected social institutions. I think this is very dangerous. Much of it comes from the populist right, and it threatens democracy. Rather than bringing institutions down, therefore, the challenge is to reform them from within. I think this can be done, but only from the ‘bottom up’ – that is, from the initiative of those working at the coalface: teachers, carers, and so on. The principal obstacles, coming from above, are professionalisation and managerialism. Professionalisation invariably places technical expertise before the vocational duty of care; managerialism places efficiency and value for money before trust and community. Going back to my answer to your first question about the meaning of a life, what has been lost from our institutions is precisely the *amor mundi*, the love for the world, which make of a life something more than a curriculum vitae. The people who work in institutions may still have it, but it has fallen through the institutions themselves like water through a sieve.

### Correspondence

*The concept of «correspondence» is essential in your discussions on education*,* skill*,* perception*,* and creativity. As you define it*,* correspondence is not about sending messages back and forth but about co-evolution and mutual unfolding of beings and their worlds. This idea is central to your critique of how conventional anthropology often depicts people and their environments as separate entities interacting mechanically. Correspondence implies a way of learning*,* knowing*,* and being that is participatory and experiential*,* grounded in life’s movement*,* flow*,* and transformation. It suggests that knowledge and understanding emerge from actively engaging in the world rather than observing it from a distance. This approach challenges the dichotomies between subject and object*,* culture and nature*,* highlighting instead the entangled and emergent qualities of life processes.*

*In The Life of Lines *(2015),* you explore how lives intertwine and unfold in correspondence*,* creating a meshwork of interwoven paths and stories. In your latest book*,* The Rise and Fall of Generation Now (*Ingold, 2024*)*,* you apply these ideas of lines and correspondence to explore different views of what a generation is and how generations connect. And you try to show that* how *we think about generations*,* that is*,* what concepts and ideas we use when we speak of generations*,* has profound consequences for what shape we allow the future to have.*

*In the field of relationally oriented therapies*,* the idea that people interact as “systems” is popular. For instance*,* we speak of systemic therapies to refer to therapeutic practices expressive of a relational ontology of mental or psychological phenomena. Drawing genograms*,* for instance*,* is a popular activity among family therapists. Your concept of correspondence represents a critique of “relation” as a sensitising concept*,* and the kind of ontology that the idea of systems invites. Could you explain what you mean by that?*

Systems thinking does, I believe, imply a different view of ‘the relation’ from the one I have been advocating. The system is assembled from moving parts, and the relations are then of cause and effect, between one part and another. My way of thinking about relations is more musical than mechanical. They are relations that go along, in the concurrent unfolding of movements that are continually responsive to one another, like lines in a choral work or fugue. That’s what I mean by correspondence.

*Could you expand a bit on what role the concepts we use to think with have for what futures are possible and not?*

If we think chronologically, then past, present and future are arrayed in a linear sequence. Viewed from the present, the future is a *project*; the past is an *abject*, over and done with. But imagine, instead, that you are walking with your children along a path that, when you were young, you walked with your parents – and which your parents, when they were young, walked with theirs (your grandparents). Then, actually, your future lies in following these ancestral ways. To lose the ways of the past is to lose your future as well.

*We also think that your distinction between network (which is a highly used concept/metaphor within relationally oriented practices in our field) and meshwork is helpful. Could you elaborate a bit about that distinction and its possible consequences for how we as professionals may engage in people’s lives?*

The distinction between meshwork and network follows from what I said earlier about musical versus mechanical thinking. Instead of speaking of the relation as a line between two things (or persons), we speak of each thing (or person) as a line, and of the relation as their intertwining. This does make a difference when we come to engage with people’s lives. With the meshwork, each life unfolds along its own line (or lines). But with the network, each lifeline is compressed into a point. Hence to engage with another person, thus compressed, is not to bind your life with theirs. It is to interact with them, but not to correspond. There is no room for attention and response; only for cause and effect.

### The Bio-Social Domain

*The psy field*,* with the medical discipline psychiatry at its frontier*,* has tended to regard mental health difficulties as individual and biologically based illnesses. As a reaction to such biologization*,* attention to the body has been replaced with attention to the psychological or social domain. However*,* this seems to have resulted in a neglect of the body because “body” is often equated with the body as “bio-mass”. In a recent paper*,* Brinkmann and colleagues (*Brinkmann et al., 2023) *make use of your work to argue that biology should be re-introduced and even re-enforced in the psy field. What they call for*,* however*,* is “another kind of biologization”: an ecology-based biology and a situational life science. Such a biologization*,* they argue*,* is needed not only to transcend a reductionist view of the body but also to prevent “mentalism” in the psy field. In this context*,* we think your concept of bio-social becomings as an alternative to human beings (*Ingold, 2013b*) has great potential for bringing our field into new lines of thinking and practices.*

*Mental health care is not your field; however*,* to what extent*,* and in what ways*,* do you think your way of bringing the body/biology into social anthropology may also speak to a field such as mental health?*

I agree with Brinkmann’s call for an alternative biology, which is essentially relational and processual. This allows us to do away with any dichotomy between the biological and the social. In recent years, however, I have also endeavoured to take some of the weight off the body, against what sometimes looks like an obsession in anthropology, as in other social sciences, with the embodiment of everything. This obsession has its roots in western ways of thinking, which are not universally shared. Non-western ontologies tend to place as much if not greater emphasis on breath, often associated with the domain of mind, soul or spirit. I think we can get beyond the awkward dichotomisation of body and spirit by focusing, in the first place, on the idea of animacy or ‘aliveness’. We can then ask questions about how creatures of different kinds, whether human or non-human, are alive to the world. And these are biological questions.

*We also notice that the term you introduce is not “bio-psycho-social becoming”*,* as one might expect. Is the omission of the “psycho-” deliberate and*,* if so*,* what are your reasons for leaving it out?*

If the biological and social dimensions of human being were separate and complementary, as was supposed in classical social anthropology, then you might still need to invoke the ‘psycho’ as a kind of glue that sticks them together. It provides for an individual mind at the point of intersection between the individual (biological) body and the collective (social) mind. But if we collapse the distinction between biological and social domains, as in the idea of biosocial becoming, then we no longer need the glue! So, the ‘psycho’ disappears. Admittedly, ‘biosocial’ is not a good word. It is a hybrid, which gives the impression that the bio- and the socio- are two, originally separate substances that have somehow to be grafted together. But for want of any alternative, we seem to be stuck with it. What we really mean, of course, is that from a relational perspective, life itself is social through and through. Years ago (Ingold, 2000: 4), I coined the phrase ‘organism-person’ to get across the same idea: not that the being is a compound of the two – one part organism, one part person – but that organism and person are one and the same.

### Surface Visions

*Lately*,* we have been working with the idea that a shift in the field of mental health is needed*,* namely a transition from directing attention towards the* inner mental “landscape” *of each person to the* outer real landscapes *in which we live in various places together with others (*Hope et al., 2024). *Here*,* we have also been inspired by your work. In an early paper (*Ingold, 1993*)*,* you point out how explorations of human living must consider the landscapes in which those lives are lived. A landscape is where life unfolds. As you put it: “Through living in it*,* the landscape becomes a part of us*,* just as we are part of it” (p. 61). Such a landscape is lived and discovered through “bodily movement from one place to the other*,* and the gradually changing vistas along the route” (p. 61).*

*What are your thoughts on how your anthropological view on human living within a landscape can be brought into the psy field to broaden how we understand and form practices to help people with their distress and difficulties?*

Maybe the concept of taskscape, which I introduced in the same paper to which you refer, might help here. The taskscape is an array of activities that people carry on in relation to one another. In fact, I introduced the concept to show why, ultimately, we don’t need it. That is to say, once we recognise that every component of what we call a landscape is doing its thing, as a movement in time, and not just its human or animal inhabitants, then taskscape and landscape merge into one. But the idea of task is important, since it signifies an activity that you carry out because it falls to you to do it, by virtue of your involvement in a community of relations. It is an activity that you owe rather than own. In this sense, helping someone in distress is a task. But it is a task discharged within the wider taskscape, within which both you and the person you help find your own existence.

*Your work*,* particularly the paper “Surface Visions” (*Ingold, 2017*)*,* has inspired us to pay attention to the surface as the domain of human social life. You put it this way*:

*“But what if there is nothing underneath? What if surfaces are the real sites for the generation of meaning? Then*,* by mining them*,* excavating them*,* or clearing them away*,* we may*,* in fact*,* be destroying precisely what we seek to find*,* and that lies under our very noses*,* convinced as we are that the truth can never be on the surface but somewhere deeper down. (…) Is it not*,* precisely*,* in such relations of interfaciality that social life is lived? If that is so*,* then the turn to surfaces is nothing less than a restoration: the restoration of social life itself”* (Ingold, 2017, pp. 100 and 105).

*We believe that how you give attention to the surface as that domain of social life also speaks to where attention could*,* or should*,* be directed when it comes to helping people with difficulties in their lives (as is the purpose of mental health services). However*,* the surface is often overlooked in many versions of psychological theory and method*,* as simply a screen where symptoms appear.*

*Do you agree that such a shift of attention towards the surface could lead to new ways of developing practices in mental health? And what*,* in your view*,* might such new practices be about?*

I suppose it could help, in so far as it would encourage us to take what people say, and their expressions in saying it, literally at face value, rather than always treating it as a symptom of something else which lies deeper down, and that it is the job of the practitioner to extract. Again, it is about listening to what people are telling us, instead of listening for what it tells about them. I recall a training session, at the university where I used to work, on how to conduct staff appraisals. The expert trainer told us that we should concentrate on the ‘body language’ of the appraisee, for what signals it might give off to reveal what they are ‘really’ thinking. I was furious! What we were being recommended to do was to substitute, for a two-way conversation with a colleague, a form of interrogation in which the niceties of conversation are but a smokescreen for the covert extraction of evidence, which could be used for or against the individual in question in the determination of his or her prospects. I dare say that the same thing happens in the mental health field, in the expert appraisal of patients. I think it is wrong on all counts – intellectual, methodological, moral and ethical.

### The Influence from Deleuze and Guattari

*In Lines - A Brief History (*Ingold, 2007*)*,* and Being Alive (*Ingold, 2011*)*,* you explicitly build on the work of Deleuze (with and without Guattari) to develop what is almost a phenomenology of the line. In that book and several other articles and books*,* you bring up the contrast between thinking with the concept of being and that of becoming. In his book Difference and Repetition (*Deleuze, 1994*)*,* Deleuze argues that every identity has difference within it*,* and that difference precedes identity.*

In which way are there similarities and differences between you and Deleuze in describing becoming?

That’s as hard to answer as it is to figure out exactly what Deleuze means by anything! Usually what happens with me is that I think something out for myself and only then turn to Deleuze. What I have thought out for myself then provides a kind of key, which unlocks the meaning of what Deleuze is saying. ‘Oh, so that’s what he means’, I say to myself. ‘Now I get it’ – which implies, of course, that he thought of it before I did. And mostly, this is the case. But if my thinking provides the key to unlock his, then whatever differences there might be between us are occluded. If we are on the same wavelength, it is largely, I think, because we share the same inspiration in the philosophy of Henri Bergson. I read Bergson in the 1980s, and was blown away by it, although it was deeply unfashionable at the time. Deleuze wrote his *Bergsonism* in 1988, though of course I didn’t come across it until much later. For a long time, I dismissed all of Deleuze and Guattari as so much incomprehensible gobbledygook, of the kind for which Francophone intellectuals are notorious. It was a bit galling to discover, then, that they had actually already said, in their own inimitable way, much of what was to come in my *Lines* book. But they said it differently. *A Thousand Plateaus* (Deleuze & Guattari, 2004) is like an immense landscape. You can take a walk through it from wherever you want to wherever else, and however many times you do, there are always new things to notice which you hadn’t noticed before. What my writing gives you is not the landscape as a whole but some of the walks I have taken through it, and what I’ve noticed along the way.

*Deleuze and Guattari claim that there are two different forms of science*,* minor and major*,* and that both have their rightful place in science. What are your thoughts about that?*

This is an example of one of those things I thought out for myself (though very much influenced by Bergson) before I encountered it in Deleuze and Guattari. Had I not done so, I would not have had a clue as to what they were talking about! Later, I read the work of Michel Serres, and discovered that the idea really came from him. Serres (in *The Birth of Physics*, 2000) was writing about the great work of the Roman poet-philosopher Titus Lucretius Carus, *The Nature of Things*, arguing that we would have a completely different history of science if his way of thinking had not been trumped by the scientific revolutions of the early modern period (with Bacon, Galileo, and the like). I am in sympathy with the argument, and I think it makes sense of a lot of the conundrums in contemporary cosmology that mainstream physics seems unable to express without going round in circles.

*Can “difference-in-itself” (Deleuze’s concept) help us formulate a theory of practice that is sensitive to the uniqueness of persons*,* situations*,* events and contexts and that reduces the risk of marginalising persons that do not fit the knowledge/“evidence-based” categories and methods (“the tyranny of selection”)?*

Yes, it can, above all by prizing the concept of difference away from otherness. Deleuze’s concept is much closer to the difference of differential calculus – a tangential deviation rather than a categorical division. As such, it focuses on processes of differentiation rather than their outcomes. It shows how difference is generated in the very process of going along together. I would say that this is generative of singularity rather than uniqueness. (In mathematics, a singularity is something like a cusp, or a point of inflection in a curve.) Uniqueness tends to focus on things in isolation, by comparison to other things, whereas singularity is about the shape of a movement. But the source of marginalisation lies precisely in turning differentiation from togetherness into otherness. This is what happens, as Deleuze shows us, when we allow the process (of becoming) to be overtaken by its outcomes.

### Education

*As educators*,* our students train to be therapists or mental health workers. Increasingly*,* neoliberal ideology keeps formulating academia as a system for transmitting knowledge; we provide students with information and concrete methods for therapeutic work. At the same time*,* we want students to evolve into sensitive*,* respectful and attentive practitioners. In your writing (*Ingold, 2018b*)*,* you are critical of the idea that knowledge and understanding boil down to information that can be transmitted*,* and you argue that education is about leading a life – attending to things rather than acquiring the knowledge that absolves us of the need to do so; it is about exposure rather than immunisation (*Ingold, 2018b, p. *ix).*

*How does your concept of ‘education as a way of life’ differ from conventional views of education*,* and what are the implications for educational practice?*

I have been inspired by the philosopher Jan Masschelein (2010), who sees education as a way of ‘leading out’ (from the Latin *ex*, ‘out’, plus *ducere*, ‘to lead’). It leads us out into the world, forcing us to relinquish established positions or standpoints, and to allow things into our presence just as they are, so that we can attend to them and learn from them. Masschelein calls this ‘poor pedagogy’, meaning that it has no body of authorised knowledge to transmit, nor any method for doing so. And rather than arming us with knowledge to shore up our defences against a hostile world, it disarms us, leaving us vulnerable but also open to what we can learn from paying attention. This is of course the very opposite of the kind of education that has a rich body of knowledge to transmit, and teaches us in the competitive arts of attack and defence in arguing or holding a position. Biesta (2013) has proposed much the same contrast in terms of an opposition between ‘weak’ and ‘strong’ education. Neither Masschelein nor Biesta is saying that education should *only* be poor and weak, rather than rich and strong. It is rather a question of the balance between the two, which has tipped disastrously to the rich/strong at the expense of the poor/weak.

*Thinking with your vocabulary of “human correspondence” and the potential of “the posture of leaning over” in education (*Ingold, 2024, p *98)*,* do you have any thoughts on how your concept of education can be applied to mental health education?*

I imagine that in training therapists or mental health workers, you need an education that is both strong and weak, or a pedagogy that is both rich and poor. The problem is to find the right balance between the two. But as I have no experience in this area, I think this is a question for you to answer, not me!

## Afterthoughts

In this interview, we have engaged the anthropologist Tim Ingold in a conversation about his take on anthropology and how it might relate to and illuminate some foundational questions, challenges and stakes in mental health - both as a field of academic enquiry, as education, and as a domain of professional practice. The interview is not an attempt to offer a how-to guide on using Ingold’s ideas for these purposes. Instead, we hope to inspire further discussions about how these ideas can come into play in mental health and the psy disciplines – for practitioners, researchers, educators, students, along with all of us who undergo whatever it is we refer to when we refer to mental health.

One way to begin such a discussion is through the notion of poor or weak practice as an invitation to turn towards the world, making us susceptible to what we can learn from paying attention. The question of how to conceive the object of mental health practices that have characterised the field since the controversies between Jaspers and Kraepelin in the early 20th Century (Ghaemi, 2009) has perhaps inserted a wedge of inward-looking self-doubt at the heart of the mental health field itself. Although the motivation for these controversies must be assumed to be precisely a desire to make sure we take in the reality of human suffering most correctly and helpfully, the many and lasting controversies over the nature of mental disorders and the appropriateness of the «medical model» itself (Matthews, 2007; Pickering, 2005; see also Boyle & Johnston, 2020) could be seen as having diverted attention from what is really at stake. The very activity of looking for ever-better models, and ever-better arguments for championing these models, is perhaps part of the procedure of immunisation that, according to Ingold, is effected by methodology, the aim of which is to rule out any possible influences arising from our correspondences with the people we seek to care for, and the issues we seek to investigate (Bøe et al., 2018).

In the ‘Return to Reality’ research initiative, we seek ways to shift from theoretical models that distance and obscure mental health phenomena and practices as objects of study. Instead, we look for ways, and concepts, that allow us to engage with these phenomena as parts of the world – our common world, the one world in which life is real. What Ingold tries to convey through the idea of ‘correspondence’, as we understand it, is that listening to the world and responding with care, sensitivity, and judgment can help restore our kinship with the earth and its inhabitants. This seems more pertinent than ever in these times of ecological and humanitarian crisis. In a collection of essays and reflections entitled “Correspondences”, Ingold (2020) likens the process of corresponding to how we used to correspond through handwritten letters. Here, thoughts and impressions inscribed in writing through the movement of the hand, are sent off along a trail to a recipient to be picked up, taken in through the eyes and digested (in the “heart” perhaps), before a response is formulated through the arm and the pen on a paper and sent back along the same trail. And so it goes, on and on, weaving lives together.

However, we do not mean to suggest that Ingold resists theory – on the contrary, he is as eager to digest theory as he is to produce it himself. Nor are we sceptical of theory in the Return to Reality project; for us, indeed, theory is the very food of curiosity, enabling us to correspond with the world in novel, often unforeseen and surprising ways.

The educational thinker Gert Biesta, in an earlier research interview (Bertelsen et al., 2023), emphasises the importance of a world-centred approach to education, by contrast to curriculum-centred or student-centred approaches. Education, Biesta argues, should be about not just acquiring knowledge or skills but engaging with the world in a meaningful way. This involves recognising that the world asks something from us and that we are responsible for responding to it. To Biesta, the concept of subjectification, which is about becoming a subject capable of navigating the world, lies at the heart of what education is for. As a process of education, subjectification involves engaging with one’s freedom and taking responsibility for one’s actions. Thus, subjectification is not just about conforming to social norms or acquiring qualifications but about developing a sense of agency and responsibility. This involves both recognising the limits of our desires and actions, and being open to what the world demands of us.

In his own writings on education, Ingold has been significantly influenced by Biesta’s thinking (Ingold, 2018b). Although the two thinkers differ in the scope and subject matter of their respective inquiries, they share a reluctance to reduce individuals to objects of intervention. Instead, they champion an approach that involves engaging with people as subjects in their own right, and that emphasises the importance of a relational and generative engagement with the world. This means being open to what the world and others ask of us and responding in sensitive and responsible ways.

According to the World Health Organization (2022), “mental health” is an area of significant global health concern. There is no doubting the reality of the range of phenomena covered by the term. As Ingold points out above, although the principal obstacles towards finding workable ways to provide good mental health care come predominantly from above, through neoliberal technologies of professionalisation and managerialism, the responsible thing to do might not be to tear down the institutions and start anew. However, since professionalisation invariably places technical expertise before the duty of care, and managerialism places efficiency and financial concerns before trust and community, we cannot either assume that the theories and traditions of the mental health care establishment will provide sufficient guidance in the search for ways to redirect attention to the world. As Ingold states in this interview, “what has been lost from our institutions is precisely the *amor mundi*, the love for the world, which makes of a life something more than a curriculum vitae. The people who work in institutions may still have it, but it has fallen through the institutions themselves like water through a sieve.” The objective of the Return to Reality project is to look for new ways of engaging with the world as mental health professionals, relying on the idea that Ingold conveys here – namely, that all of us working in different mental health institutions are really looking for ways to care for the world.

In this research interview, we have engaged with the anthropologist Tim Ingold, to jointly explore how some of his ideas can enrich the way we conceive of, and *do*, the work of mental health practice. We hope this may inspire others to pursue their own correspondences.

## Data Availability

No datasets were generated or analysed during the current study.
